# Challenges in Nuclear Medicine: Innovative Theranostic Tools for Personalized Medicine

**DOI:** 10.3389/fmed.2014.00016

**Published:** 2014-07-03

**Authors:** Françoise Kraeber-Bodéré, Jacques Barbet

**Affiliations:** ^1^Nuclear Medicine, University Hospital-ICO, CRCNA, INSERM U892, CNRS UMR 6299, Nantes and GIP Arronax, Saint-Herblain, France

**Keywords:** nuclear medicine, personalized medicine, PET imaging, FDG–PET, targeted therapies

Over the past few years, nuclear medicine has undergone impressive growth with the development of positron emission tomography (PET), especially using ^18^F-fluoro-deoxy-glucose (^18^FDG), and new approaches in targeted radionuclide therapy. These developments pave the way for personalized medicine by offering practical solutions, especially in oncology, neurology, and cardiology. Novel radiopharmaceuticals targeting relevant biomarkers are powerful patient selection tools for patients who may benefit from targeted therapies, and for early therapeutic response assessment. Moreover, once labeled with beta- or alpha-emitters, radiopharmaceuticals targeting relevant molecular markers expressed by different solid tumors, and hemopathies can be used for radionuclide therapy. The final objective here is to eradicate residual cancer disease by using cytotoxic mechanisms complementary to those of “non-radioactive” therapies. PET imaging and targeted radionuclide therapy then come together in the context of the theranostic approach to adapt injected activity for personalized therapy.

^18^FDG–PET demonstrates the high accuracy and the clinical benefits of non-invasive whole-body imaging. It is used in clinical practice for initial staging and therapy evaluation in several solid tumors and hemopathies. ^18^FDG–PET is also applied outside oncology to explore dementia, assess myocardial viability, or detect infectious and inflammatory processes. However, ^18^FDG is not a specific tracer. For example, in solid tumor or lymphoma assessment, ^18^FDG–PET does not distinguish tumors from inflammation, inducing false positive results, especially after therapies inducing inflammatory or immune reactions. New radiopharmaceuticals are needed to better characterize pathologic processes and to predict and assess response to therapy (1).

In oncology, the development of ^18^FDG–PET is ongoing, particularly for therapy response assessment. Image acquisition and analysis protocols are being further standardized to improve diagnostic accuracy (2). For example, specific criteria have been elaborated to assess lymphoma or solid tumor response to therapy (2–4). For tumors with low avidity for ^18^FDG, other ^18^F-labeled compounds are being proposed (e.g., ^18^F-choline in prostate cancer or hepatocellular carcinoma) (5). In addition, phenotype-specific tracers are needed for theranostic applications: ^68^Ga-labeled somatostatin analogues improve image quality in neuroendocrine tumors in comparison to ^111^In-octreotide available in clinical practice, with a significant clinical impact. Several ^68^Ga-labeled somatostatin analogues with variable performances have been evaluated (6–8). These novel imaging radiopharmaceuticals are particularly interesting because cold- and radio-labeled somatostatin analogues are efficient for therapy of neuroendocrine tumors. Numerous peptides, targeting other receptors, are in development. Radiolabeled bombesin analogues show promise for prostate cancer (9, 10). Radiolabeled RDG peptides targeting the α_v_β_3_ integrin have potential in a large spectrum of indications in the field of oncology but also in cardiovascular diseases (11, 12).

Targeted therapies, including monoclonal antibodies (MAbs), experience a considerable growth in cancer management. MAbs are also promising vectors for theranostic approaches, to better identify patients who will respond and to monitor responses (13). Based on immuno-PET, treatment strategies could be tailored for individual patients before administering expensive and potentially toxic therapies. Until now, only invasive methods such as biopsy with immunohistochemistry analysis could identify patients who have the highest chance of response to antibody-based therapy. Immuno-PET can offer a non-invasive solution to quantitatively assess target expression. For example, anti-HER2 therapeutic agents are most effective in patients who have *HER2*-positive breast cancer as determined by immunohistochemistry. It has been proven that MAbs labeled with ^68^Ga, ^64^Cu, or ^89^Zr could non-invasively identify lesions that are likely to respond to therapy (14–16). Immuno-PET is also a powerful innovation to improve knowledge about the efficacy and *in vivo* behavior of MAbs.

Imaging plays an increasing role in the development of new drugs by pharmaceutical companies: *in vivo* imaging constitutes an effective solution for the rapid assessment of drug candidates, which may be radiolabeled to monitor their pharmacokinetics and biodistribution during pre-clinical and early clinical phases. Alternatively, molecular imaging using radiopharmaceuticals, combined with biomarkers, gives information about the quantitative variation of molecular targets during treatments. Indeed, if ^18^FDG–PET can be used (17), radiolabeled tyrosine kinase inhibitors (TKI) analogues are also developed to evaluate TKI efficacy in clinical trials by PET imaging (18).

Nuclear medicine is also advancing cancer therapy. Clear examples are the treatment of non-Hodgkin’s lymphoma by anti-CD20 or CD22 MAbs labeled with yttrium-90 (19, 20) and of neuroendocrine tumors by somatostatin analogues labeled with yttrium-90 or lutetium-177 (21, 22). However, many other tumors must be addressed and promising results have been reported in prostate cancer and colorectal carcinoma (23, 24). These treatments are generally not curative. More efficient radiopharmaceuticals and more toxic radionuclides, such as alpha-emitters, as well as combination therapy or multiple injection protocols, seldom studied yet in the field of targeted radionuclide therapy, should be developed with the objective of killing the last tumor cell, which is most probably a cancer stem cell (25). Efficacy of alpha-therapy has been recently shown in a pre-clinical model of multiple myeloma using anti-syndecan 1 MAbs labeled with the alpha-emitter ^213^Bi (26). Fractionated radioimmunotherapy with ^90^Y-clivatuzumab and low-dose gemcitabine demonstrated activity in advanced pancreatic cancer in a phase I clinical trial (27). Pre-targeting using bispecific antibodies and radiolabeled bivalent hapten-peptides showed promising results in CEA-positive tumors (28, 29). Predictive patient dosimetry can be obtained using a test dose to adapt injected therapeutic activity to each patient pharmacokinetics and biodistribution (30).

The development of new radiopharmaceuticals requires radionuclides with physical half-lives that match the vector biological half-lives. For imaging purposes, short half-life PET emitters such as ^68^Ga and ^18^F are well suited for labeling drugs with fast distribution kinetics. The short half-life reduces exposure and allows for pharmacological studies of receptor occupancy, for instance. For other vectors that reach their target more slowly, such as antibodies, radionuclides with longer half-lives are needed (^64^Cu, ^44/44m^Sc, ^89^Zr) (31). In addition, couples of β^+^/β^−^isotopes (e.g., ^64^Cu and ^67^Cu) are of particular interest for PET/therapy radiopharmaceuticals. For therapeutic purposes, the penetration path-length of the radioactive emission should match the size of the targeted tumor. Yttrium-90, with its long-range beta^−^ emission, is better suited to bulky diseases. However, promising results have been observed using ^90^Y-radioimmunotherapy in the consolidation setting in responding lymphoma patients (partial or complete responders) after induction therapy (32). Radionuclide such as ^131^I, ^177^Lu, or ^67^Cu with shorter-range beta^−^ emissions should be more favorable in the minimal residual disease setting. Alpha particle emitters offer the theoretical possibility to kill isolated tumor cells and microscopic clusters of tumor cells because of the short tracks of alpha particles (25). For alpha-emitters, ^213^Bi is available through a ^225^Ac/^213^Bi generator. Its short half-life (46 min) and cost are potential limitations. Despite its complex chemistry, ^211^At may be a better candidate for alpha-therapy due to its longer half-life (7.2 h) and other radionuclides with complex radioactive decay schemes are proposed (^212^Pb, ^225^Ac, ^227^Th) (33).

In the decades to come, we anticipate that new radiopharmaceuticals targeting specific biomarkers will be developed for early diagnosis and prognostic prediction, and to adapt therapies to each patient in oncology, as briefly described above, but also in other domains, such as neurology or cardiology. We believe that PET represents a promising tool for molecular diagnosis, providing non-invasive whole-body cartography of biomarkers, complementary to biological testing. Moreover, we believe that targeted radionuclide therapy will develop, especially in multimodality strategies.

The goal of *Nuclear Medicine* is to be a forum for publication of pre-clinical and clinical findings that assess the role of new and existing radiopharmaceuticals in the molecular characterization of disease before and after treatment, with the ultimate goal of personalized patient management. This section is also open to contributions in the fields of radiopharmaceutical discovery, new radionuclide therapy procedures that use innovative radionuclides or radiopharmaceuticals, multimodality strategies and original methods of image analysis, quantification, and dosimetric evaluation.

## Conflict of Interest Statement

The authors declare that the research was conducted in the absence of any commercial or financial relationships that could be construed as a potential conflict of interest.
